# Perceptions of postgraduate family medicine supervision at decentralised training sites, South Africa

**DOI:** 10.4102/phcfm.v14i1.3111

**Published:** 2022-03-14

**Authors:** Neetha J. Erumeda, Louis S. Jenkins, Ann Z. George

**Affiliations:** 1 Department of Family Medicine and Primary Care, Faculty of Health Sciences, University of the Witwatersrand, Johannesburg, South Africa; 2 Gauteng Department of Health, Ekurhuleni Health District Services, Germiston, South Africa; 3 Division of Family Medicine and Primary Care, Faculty of Health Sciences, Stellenbosch University, Cape Town, South Africa; 4 Western Cape Department of Health, Eden District, George Hospital, George, South Africa; 5 Centre of Health Science Education, Faculty of Health Sciences, University of the Witwatersrand, Johannesburg, South Africa

**Keywords:** clinical supervision, decentralised clinical training, educational supervision, family physician, family medicine registrar, postgraduate training, workplace-based learning

## Abstract

**Background:**

Specialist training in family medicine (FM) is growing rapidly in sub-Saharan Africa. The strong emphasis on workplace-based learning for speciality training makes it vital to gain in-depth insights into registrar supervision. Previous studies have explored aspects of supervision at decentralised sites in high-income countries, however, little is known about the benefits and constraints of decentralised postgraduate supervision in low- to middle-income countries, especially in Africa.

**Aim:**

This study aimed to explore family physicians’ and registrars’ perceptions of the strengths and challenges of clinical and educational supervision across decentralised training sites.

**Setting:**

The study was conducted across two provinces at five decentralised training sites affiliated with the University of the Witwatersrand, Johannesburg.

**Methods:**

This qualitative study involved semi-structured interviews with a purposive sample of 11 FPs and 11 registrars. The data were thematically analysed.

**Results:**

Two of the four themes identified, ‘supervision is context-specific and supervisor-dependent’, and ‘the nature of engagement matters’, involved strengths and challenges. The other two, ‘supervision is not ideal’ and ‘the training environment is challenging’, focussed on challenges.

**Conclusion:**

Supervisors and registrars described the postgraduate FM supervision as context-specific and supervisor-dependent. Supervisors displayed good clinical-teacher characteristics and supervisory relationships. However, several challenges, including registrars’ workload, resource shortages and a lack of standardisation across training sites, need to be addressed. Regular faculty development is essential for supervisors to be aware of relevant aspects of, and current trends in, postgraduate training.

## Background

In 2013, the World Health Organization advocated expanding health professionals’ training from academic centres to decentralised training platforms in order to address communities, and nations’ priority health concerns.^1,2^ Decentralised clinical training (DCT) refers to training at sites away from tertiary centres for six months or more in the context of primary healthcare (PHC) clinics, community, district or regional hospitals, or general practice settings.^3^ While DCT is well established in high-income countries (HIC), such as Australia, the United Kingdom and Canada,^4^ it is a relatively new concept in low- and middle-income countries (LMIC), such as South Africa (SA) and other sub-Saharan nations.^3,4,5,6^ Decentralised clinical training offers trainees more hands-on experience of clinical and procedural skills during longer longitudinal rotations. It also provides flexible curricula typical of postgraduate medical training.^3,7^ Despite these affordances, DCT is affected by factors more likely to prevail in LMIC, including a lack of resources and comprehensive support. Training at DCT sites has been shown to improve healthcare provision across communities; however, DCT needs to be optimised in order to ensure that the priority health concerns of LMIC are met.^3,4,8,9^

Supervision of postgraduate trainees is a vital component of DCT, which involves workplace-based learning (WBL). Workplace-based learning is driven by the needs of the workplace, allowing trainees access to training and development while fulfilling other roles and responsibilities.^10^ Workplace-based learning prioritises informal learning opportunities, maximising cognitive apprenticeship models,^11^ role modelling,^12^ communities of practice^13^ and situated learning.^10,14^ Situated learning refers to the learning that occurs by participation in and collaboration with other professionals in a community of practice.^14^ The informal learning opportunities associated with WBL are less valued than formal training sessions, as they are regarded as disorganised, opportunistic and lacking in formal educational rigour or structure.^10,15,16^ Despite the negative view of informal training, the informal training opportunities in WBL make possible various postgraduate training methods, all of which emphasise the importance of the adequate training and supervision of trainees in the workplace.^16,17^ These methods include supervised participation in practice, including entrustment and support-seeking; mutual observation of practice, including monitoring and modelling; and dialogue during practice, including meaning-making and feedback.^12^ Despite the vital role of supervision in DCT, existing studies have not explored the informal learning opportunities in WBL adequately in the context of LMIC.

There are two types of supervision in WBL. Educational supervision occurs in recognised training contexts, while clinical supervision is less formal and occurs at the patient bedside or in the primary care context.^18^ Educational supervision involves establishing trainees’ learning needs and reviewing their progress, identifying educational opportunities and compiling learning portfolios.^18,19^ The role of an educational supervisor focuses on developing a good relationship with trainees and facilitating their professional, personal and educational progress, including being a good listener, assessing trainees’ current knowledge, skills and attitudes, and providing feedback.^19^ The role of a clinical supervisor includes providing supervision tailored to trainee competence and experience,^16^ being available to trainees, providing regular feedback and responding timely to their learning needs.^20^ The encounters of clinical supervision range from daily developmental conversations^20^ on clinical management to reflective case-based discussions in hospital wards or clinics.^18^

Effective supervision in the workplace is a multi-faceted concept requiring training on beneficial teacher characteristics and appropriate role-model behaviour. Supervisors should display excellent clinical knowledge and skills, professionalism^21,22^ and teamwork, and encourage students to reflect on their practice and balance their time and professional and personal lives.^12,23,24,25^ Supervisors should know what constitutes a good supervisor–trainee relationship, that is, showing evidence of a bond, trust, agreement of supervisory goals and openness.^26^ Supervision requires supervisors to have current knowledge and continually improve their clinical reasoning in the context of challenging heavy clinical workloads.^26,27^ These supervisor characteristics also provide trainees with opportunities for self-reflection,^26^ which is essential for the self-regulated learning required during WBL.^28^

Postgraduate family medicine (FM) training at DCT sites in African countries is in varying phases of development.^9,29,30,31^ Family medicine was recognised as a speciality in SA in 2007.^32^ Before this, many other African countries (like Uganda and Kenya) had established academic FM decentralised-training complexes to strengthen their PHC services.^9,33,34^ However long FM training has been taking place in these countries, there is a critical need for trained and competent family physicians (FPs) to service these training complexes. Despite the roles of FPs and the training outcomes in sub-Saharan Africa having been identified,^9,35,36,37^ Family medicine training is disadvantaged by a lack of a shared understanding of FM on the part of stakeholders (general practitioners, specialists and policymakers), FP shortages, a lack of existing policies and inadequate funding.^4,9,38^

Decentralised FM training in SA differs from training in HIC. While FM training in HIC occurs in private general practice settings,^39,40,41^ in SA, registrar training (training for South African, postgraduate, medical trainees) is mainly rotational across PHC clinics and public hospitals. At these sites, FPs, specialists and registrars in some clinical departments and medical officers (MO) who have had no formal training in supervision play a critical role in training students, interns and junior doctors.^42,43^ The public-service setting of FM training in SA aggravates difficulties associated with supervision. The challenges include a lack of meaningful feedback or direct clinical contact as part of career progression by some supervisors and heavy clinical and academic workloads for supervisors.^7,44,45^ Some of the strengths of DCT in SA, as identified in previous studies, include the characteristics of the learning environment, supervisors’ competence and teaching styles, the use of learning portfolios, the quantity and quality of learning conversations, and opportunities for role modelling.^44,45^

At the University of the Witwatersrand (Wits University), one of nine South African universities offering a 3-year full-time postgraduate FM programme, the DCT sites are spread over five districts in two provinces. Registrar training in these districts takes place in both clinical and non-clinical contexts. The clinical context comprises the peri-urban or rural PHC or community health centre or district and regional hospitals, depending on the district. Community health centres are 24-h nurse-run and doctor-supported facilities. District hospitals or level-1 hospitals are categorised into small, medium or large depending on the number of beds, with limited specialist medical services and serviced by MO and FPs.^46^ Regional hospitals are larger hospitals (200–800 beds), providing 24-h services offering specialist services across various disciplines.^46^ The non-clinical context refers to district regional training centres (RTC) based within clinics or district hospitals.

Given the potential for DCT to improve healthcare provision and medical education in SA, there is a lack of in-depth exploration of supervision, supervisory roles and the supervisor–trainee context interactions in sub-Saharan Africa. The purpose of this study was to explore FPs’ and registrars’ perceptions of the strengths and challenges of postgraduate supervision in clinical and non-clinical contexts at Wits University. These findings could contribute to a more effective and standardised local programme that could benefit other universities nationally and in other sub-Saharan African countries.

## Methods

### Study design and setting

The reported study forms the qualitative component of a broader convergent mixed-methods evaluation of the postgraduate FM, decentralised-training programme at Wits University. The intent of convergent mixed-methods designs is to integrate qualitative and quantitative data in order to enhance understanding of a phenomenon of interest.^47^ The qualitative component of the study used an inductive interpretative approach^48^ to explore FM supervisors’ and registrars’ perceptions of postgraduate supervision in the FM, decentralised-training programme. These groups of interviewees were purposively sampled because they possessed the characteristics being sought and could provide an in-depth understanding of the phenomenon of interest.^49^

Four of the operational districts – Ekurhuleni, Johannesburg Metro, Sedibeng and the West Rand – are situated in Gauteng province, with the Dr Kenneth Kaunda district located in the North West province. Family physicians responsible for supervising the registrars at the clinics and the RTC will be referred to as *FP supervisors*. Any non-FM doctor who supervises the registrar during hospital rotations is referred to as *non-FP supervisors. Non-FP supervisors* include specialists, registrars and senior MO in other clinical departments. The different types of supervision include case discussion between a supervisor and registrar about a patient in the consulting room or ward, facilitating presentations at RTC or clinics and leading discussions of learning portfolios with registrars. *Adequacy of supervision* refers to the extent to which supervisors fulfil the above requirements. Family physician supervisors perform as both clinical and educational supervisors in some districts, whereas in others, there is some differentiation in their roles. Non-FP supervisors participate in the supervision of the registrars on case discussions during ward rounds or while performing procedures or clinical presentations at hospitals.

### Study population and sampling

This study involved purposive sampling^49^ of FPs and FM registrars to achieve representativeness across the five training districts. The study population consisted of 20 FPs and 21 registrars. All FPs involved in registrar supervision in the five districts were eligible to participate in the study. Family Physicians who were not jointly appointed by the Department of Health and Wits University were excluded. Only registrars in their second and third years of training were invited to participate in the study. First-year registrars were excluded from the study as they were deemed not to have adequate experience of the training programme. Of the 11 FPs and 11 registrars interviewed, three FPs and three registrars were from one district, and two FPs and two registrars were from each of the other four districts.

### Data collection

Semi-structured interviews were conducted with the FPs and registrars between March and August 2020. Although initially intended to be face to face, the COVID-19 lockdown necessitated that most of the interviews (14/22) were conducted electronically using Zoom or Microsoft Teams. The interviews, which lasted for about 80–90 min, were conducted in English by the principal researcher and were audio-recorded. The FPs and registrars were asked how they supervised and were supervised, respectively, across the different training sites, and about the strengths and challenges of supervision (see Appendix 1, Interview guide). Interviews were conducted until saturation was reached and there were no new emerging themes.^50^

### Data analysis

The interview recordings were transcribed verbatim and analysed thematically using Braun and Clarke’s six-step approach.^51^ The analysis was managed in the qualitative data analysis software programme MAXQDA version 2020. After checking the fidelity of the transcripts against the recordings, the principal researcher familiarised herself with the data through several readings of the transcripts (step 1) and used open coding to develop a coding system (step 2). The initial codes were grouped into subcategories, which were then grouped into categories. Similar categories were merged into sub-themes, and then into themes (step 3) through an iterative process of consultation with the co-authors of this study. As part of the iterative process of analysing the data for patterns, we reviewed the themes (step 4) and defined them (step 5). The final step (step 6) involved writing the paper using appropriate excerpts from the data to validate the themes.

### Trustworthiness

The credibility of the study was improved through the iterative processes of coding the data and refining the codes and categories in collaboration with the co-authors, including the identification and naming of themes. Purposive sampling^49^ and data triangulation^52^ from different sets of participants, including FPs and registrars from all decentralised sites, further enhanced credibility. A detailed description of the study methods, including the sampling, inclusion and exclusion criteria, improved the transferability of the findings. An audit trail^52^ of the data management and the development of the codes, categories and themes enhanced the dependability of the study. All three authors were constantly examining and exploring perceptions of the postgraduate FM training programme, thereby augmenting confirmability.

### Ethical considerations

Ethics approval was obtained from the Human Research Ethics Committee (HREC Medical) of the University of the Witwatersrand (certificate number: M191140). In addition, permission to conduct the study was obtained from the Gauteng and North West province district research committees using the National Health Research Database (GP_201910_050). Informed consent was obtained from all participants. The research study was carried out following the Helsinki Declaration.

## Results

The characteristics of the study participants are shown in Table 1. Of the 11 FPs (FP1 – FP11), eight had more than five years of training experience and three had less than five years. Among the 11 registrars (RG 1 – RG 11) who participated, five were in their second-year and six in the third-year of training.

**TABLE 1:** Participant characteristics.

Characteristics	*n*
**Registrars**	
**Age (years)**	
≤ 30	3
31–40	6
41–50	1
51–60	1
**Gender**	
Male	5
Female	6
**Nationality**	
South Africa	7
Not South Africa	4
**Year of training**	
Year 3	6
Year 2	5
**Family physicians**	
**Age (years)**	
41–50	8
51–60	3
**Gender**	
Male	7
Female	4
**Nationality**	
South Africa	5
Not South Africa	6
**Years of training experience**	
1–5	3
> 5	8

While most FPs attended short courses, none of them had formal training in medical education. All the registrars had some level of PHC experience before joining the programme.

The findings from analysis of the perceptions of supervision by FPs and registrars were organised into four themes and 12 subthemes, as shown in Table 2. Two of the four themes, ‘supervision is context-specific and supervisor-dependent’ and ‘the nature of engagement matters’, included both strengths and challenges. The other two, ‘supervision is not ideal’ and ‘the training environment is challenging’, highlighted the challenges.

**TABLE 2:** Themes and subthemes.

Themes	Sub-themes
Supervision is context specific and supervisor dependent	FP supervision in the clinical context enables individual attention
	FP supervision in the non-clinical context provides multiple perspectives
	Non-FP supervision focuses only on the clinical training
The nature of engagement matters	Engagement is tailored to learning needs
	Supervisor characteristics impact the training
	Professionalism affects engagement
Supervision is not ideal	Time constraints among FPs
	Registrars often feel unsupervised
	Supervision is not standardised across the training sites
The training environment is challenging	Insufficient resources hinder training
	Work–life balance is challenging for registrars
	The district hospitals are perceived as hostile

FP, Family physicians.

### Supervision is context-specific and supervisor-dependent

The FPs and registrars perceived supervision to be specific to the context in which registrars are supervised with unique strengths and challenges in both clinical and non-clinical settings.

#### Family physician supervision in the clinical context enables individual attention

Most registrars valued personal contact with FPs in clinics and district hospitals. Registrars and FPs provided several examples of the types of one-on-one engagement possible when working in the same environment, such as seeing patients together and FPs observing registrars conducting consultations, which allowed them to identify learning needs. One of the registrars pointed out that a case discussion would always ensue regardless of the FP supervisor:

‘We see patients together, or where I will maybe interview a patient or she will see the patient, or I will see the patient, and we discuss the case together.’ (RG 9)

In some districts, working in the same environment allowed supervisors to model the procedures involved when conducting consultations. In other districts, FPs and registrars did not have one-on-one engagements; they consulted separately but discussed complex cases of patients when required:

‘So, you’ll be seeing your patient in your own room, he might have his own room, and then if you are stuck and you have a question, then you’ll go to him and ask’. (RG 4)

The most critical aspects of one-on-one engagement involve providing registrars with meaningful feedback on observed consultations, procedures and skills using the mini-clinical evaluation exercise and direct observation of procedural skill templates when carrying out workplace-based assessments at clinics. According to some FPs, workplace-based assessments aligned with the national examination standards, and feedback was provided accordingly. The extent of the feedback depended on the type of patient. Family physician 3 explained that some patients ‘stimulate us to go deeper and then talk (about) a lot of things with regards to diagnosis, management, theoretical things and all that’.

Some registrars, while on regional or district hospital rotations, valued individual engagements during supervisory visits by FPs. Family physicians and registrars referred to different types of FP–supervisor engagement in some districts, including conducting ward rounds and seeing patients together with registrars and case-based discussions. According to some registrars, these supervisory visits helped them to focus on more relevant skills during their rotations. At the same time, FPs described how these visits allowed them to get feedback on registrars’ progress or any issues that needed to be brought to their attention from heads of departments:

‘If there are any issues that the supervisors have raised, which has happened before, for instance, the supervisor can say, look, this person we don’t see … their attendance has been very poor, and all those kind of things, then we take it along those lines.’ (FP 11)

In districts where FP supervisors did not visit the registrars in the hospitals, FPs only received feedback from registrars about their progress during the weekly academic sessions at RTC. Unfortunately, this limited feedback denied registrars the opportunity to engage with FPs in their learning setting during rotations. Even though some registrars described meaningful individual engagements, others raised concerns over the quality of supervision, depending on the extent of the supervisors’ level of experience and their approach to teaching. For example, one registrar said:

‘How rich is the discussion? It’s very subjective [*and*] it’s also supervisor-dependent. We gain a lot from different supervisors, but it depends actually on the person who’s supervising that day.’ (RG 6)

#### Family physician supervision in the non-clinical context provides multiple perspectives

Registrars and FPs highlighted the benefits of multiple engagements and receiving input from different FPs during their RTC academic sessions. The diversity of engagements and input allowed the registrars to receive varied information, opinions and strategies from multiple FPs. According to some registrars, different supervisors focused on different areas in their feedback and had various areas of interest and expertise, which added to the robust discussions:

‘There is some benefit with having multiple opinions, and then you are able to formulate what works best for you as an individual based on different experiences that is [*sic*] shared with you’. (RG 1)

A few FPs felt that receiving feedback from more than one supervisor enabled more robust discussions compared with individual engagement at the clinics:

‘What we do at DT [*district*] 4 [*is that*] we supervise them collectively. I do it with another family physician and sometimes with two other family physicians, so we try as much as possible to give our individual input[*s*].’ (FP 4)

#### Non-family physician supervision focuses only on the clinical training

Non-FP supervision was conducted by either consultant specialists or senior registrars rotating from the university or senior or junior MO in various clinical departments. Despite this variation, both FPs and registrars valued the various clinical-training activities, which included case-based discussions, tutorials and academic sessions:

‘You basically, like if you want to discuss the patient, you go to the consultant and discuss the case, and then the consultant will advise you or come see the patient with you and discuss what needs to be done, or it will be senior registrar would come through and also like observe and examine with you. So, from that point of view you get some supervision’. (RG 10)

According to some registrars, they were invited to observe procedures or skills whenever their supervisors performed them and were allowed to practise skills repeatedly under guidance in order to achieve the expected level of competence:

‘So that’s what we agreed with them, so whoever is doing calls that day, even when I’m in OPD [*out-patient department*], if there is a procedure that needs to be done, and it’s on my objectives list, so I’ll get them to call me, I’ll go and check and see how they do it, or either I do [*it*] and then they will show me how to do it’. (RG 4)

Both participant groups perceived the nature and frequency of non-FP supervision at regional and district hospital rotations as department-dependent, variable and needing improvement regarding the extent of their involvement in training. In addition, one of the registrars perceived the lack of consultant as problematic:

‘Sometimes, when we do rotate in some of the clinical departments, not all of them have a consultant. Sometimes we find medical officers; some of them may be junior to some of us in those departments. So, it’s not all the time that we have the availability of consultants.’ (RG 5)

Despite the lack of consultants, a few FPs and registrars felt that supervision by consultants visiting from regional hospitals to district hospitals was also problematic because they were not available for outreach visits. Most registrars and FPs thought that non-FP supervisors lacked an understanding of the expectations, scope of practice and learning outcomes of FM. One of the FPs commented:

‘I’m not always certain that the specialists there are really aware of what is expected of these students, and I know that they always say they’re not becoming mini-orthopods or mini-psychiatrists’. (FP 5)

Some registrars felt that non-FP supervisor discussions with registrars were more aligned with that speciality’s clinical approach, as opposed to the biopsychosocial approach of the discipline of FM. For example, one of the registrars (RG 1) commented, ‘[*s*]ometimes when you’re doing rotations it becomes too clinical and the biopsychosocial [*approach*] – it’s, sort of, left out’.

Registrars and FPs felt that registrars were often treated like interns or MO. They could be expected to perform tasks such as taking blood or were regarded as extra workforce, which prevented them from focusing on their learning needs:

‘Most of the time, they’ll be asking, “[*w*]hat is (a) family physician?” So, it’s similar in most specialities. They, so far, don’t know exactly what is (a) family physician (and) what is expected from them to teach registrars in Family Medicine’. (RG 4)

### The nature of engagement matters

Several strengths and a few challenges that affected the nature of the engagement between registrars and FPs were identified. Firstly, the nature of engagement by FPs in clinical and non-clinical contexts is closely linked to the registrars’ learning needs. Secondly, it allows integration of the FM principles and approaches into their learning. Other subthemes mentioned included supervisors’ attitudes, the benefits of teamwork and registrars’ level of professionalism.

#### Engagement is tailored to learning needs

During FP supervisor engagement, both FPs and registrars identified that training was strongly linked to the registrars’ learning needs and gaps:

‘So you’ll have to discuss with your supervisor what you know, what you don’t know, what your learning needs are, [*and*] what gaps need to be filled to make you adequate or competent as a physician.’ (RG 1)

FPs felt that they assisted registrars in identifying their learning needs:

‘The training here is supposed to be self-directed learning based on their identified need. So, we try to give them that ability to identify what is their learning need when they go to, let’s say, like immunisation, well-baby clinic, antenatal clinic, and all that’. (FP 11)

#### Supervisor characteristics impact the training

Most registrars perceived that FP supervisors provided them with insights into patients’ comprehensive and holistic care in clinical and non-clinical contexts as commented by RG 7, ‘[*e*]ven just the way that they assess us gives us an insight into what is expected of us, as family physicians and in the examination’.

Supervisors improved the registrars’ biopsychosocial approach and clinical reasoning and bolstered their confidence in consultations during clinic sessions.

Family physicians felt that they were training registrars for the clinical role and community-oriented primary care, training students or nurses, and clinical governance roles. They kept track of whether registrars would be good FPs by observing patient interactions, examination skills, referral patterns, prescriptions, clinical record-keeping and professional behaviour. One of the FPs mentioned:

‘That’s part [*of*] the capacity builder, you know, teaching the students. So, there are other, maybe, roles of family medicine [*that*] she’s able to see or do them [*sic*] because then, in a way, she’s forced to do because it’s not just clinical’. (FP 1)

In RTC sessions, both registrars and FPs perceived that FPs helped registrars to integrate FM principles into academic presentations as explained by RG 3, ‘[*t*]hen she’s able to guide me and tell me, this is how … you’ll benefit using FM approach for anything you do in this programme’. In addition, some FPs agreed that supervisors helped registrars to focus on the practical aspects of the learning outcomes and apply them in their work environment:

‘We don’t want a situation where people are learning theoretically and not applying it on the ground. Because ultimately, it then skews the training programme to be kind of like a theoretical exercise. So, we mainly focus on the practical aspects of what they’re actually learning and their learning objectives’. (FP 11)

Some registrars perceived that while FPs promoted an FM approach, FPs displayed good clinical teacher characteristics themselves. This includes their availability, reliability, openness, honesty in communication and ability to develop a good rapport, which enhanced the nature of their engagement:

‘I think she’s a very open and honest person, and it’s easy to talk to her about issues that we are facing or things that is quite a stress for the entire group because we feel comfortable enough to talk about what’s going on’. (RG 10)

Other registrars felt that their supervisors were organised, dedicated, very willing to teach and strict about adhering to punctuality. A few observed leadership and governance skills in their supervisors with the hope of learning from them.

Most FPs regarded themselves as effective communicators who established good relationships with the registrars:

‘We have a one-on-one, we’ve built a relationship, there’s a good communication relationship between both of us. He’s able to understand me, and I’m understanding him better, which I think is important’. (FP 10)

Some FPs described their passion for teaching as their best characteristic, whereas others perceived their vast knowledge and experience as their strengths in supervision.

While good supervisor–registrar relationships enhanced engagement, a few registrars mentioned the impact of poor relationships. They felt that some supervisors needed to improve their communication skills in order to establish a better rapport with their trainees. In addition, some of the registrars expressed the need for a professional relationship with their supervisors:

‘The challenges is [*sic*] in terms of the attitude or the atmosphere of the whole learning session, whereby if you pick up, for instance, that the supervisor is not really involved in what’s going on, it becomes like … it is a routine that needs to be sorted quickly’. (RG 6)

#### Professionalism affects engagement

Some FPs described how they work and support each other as a team while engaging with registrars. Some mentioned that they asked their colleagues to fill in for them when unable to attend planned academic sessions. One of the FPs (FP 6) said, ‘I get support from other colleagues because I’m not always all the time [*sic*] there’.

Other examples of teamwork included FPs assisting the academic coordinators in planning the yearly programme. In addition, FPs in one of the districts provided registrars with feedback collectively, which helped novices learn from more experienced FPs. Registrars from the same district also observed that supervisors distributed tasks for better supervision:

‘One of the things that so far we appreciate is that tasking; I think task distribution has made things much easier for us, and we think that it has strength because it was difficult to judge the previous family physician … him being alone because of these new ones, they have more hands, so it’s easier for them to perform better’. (RG 4)

In contrast to working in teams, FPs in one of the districts perceived that training at RTC was often performed alone. They described a lack of support from their colleagues:

‘Again, it’s often only me that’s there; if someone else pitches up, that’s great, but we’ve got seven registrars, six in years one to three, so we have to do observed consults for all of them’. (FP 7)

Some FPs observed that the lack of professional behaviour and commitment by registrars affected their level of engagement. Family physicians believed that the registrars’ preparation for topic presentations was sometimes inadequate or did not meet the required standards. As explained by FP 8, ‘… and initial presentations, they tried to do them shabbily, but we quickly said, “[*n*]o, you can’t do that”’. Registrars sometimes displayed inappropriate behaviour by disregarding the FP’s instructions and sometimes arriving late or not at all. A few FPs felt that some registrars who have completed the three training years were disruptive in RTC sessions. These were fourth-year registrars who were not actively involved in the programme for the year and were excluded from the study.

Another challenge identified by FPs and specialists among registrars was their inadequate baseline skills:

‘I’ve had registrars who were put in paeds (paediatrics), and the consultant was not happy with the [*sic*] level of skills, or she negotiate[*d*] with me to put [*sic*] the registrar to do a call as [*sic*] a level of interns. Yes, so we have had challenges, specifically with registrar skills’. (FP 6)

### Supervision is not ideal

Registrars and FPs identified that supervision was negatively influenced by inadequate time because of the multiple roles and responsibilities FPs must fulfil, thus causing registrars to be often left unsupervised. Other challenges that impacted the quality of supervision included the lack of standardisation in supervision and FPs’ supervisory skills.

#### Time constraints among family physicians

Most FPs discussed how supervision is affected by their struggle to manage their multiple roles and responsibilities in clinical and non-clinical contexts. For example, some FPs kept postponing their supervising sessions at clinics or RTC because they were not available. For others, this conflicted with their administrative duties, such as unplanned meetings, clinical governance and service delivery or head of department roles:

‘It was a big challenge. The reason is the time period. Sometimes you’ve got another commitment in the sub-district in terms of [*a*] meeting or something so that [*the*] programme is sometimes postponed’. (FP 2)

Many FPs could not conduct supervisory visits during registrar rotations because of severe FP shortages or inadequate support from their superiors. Most FPs felt that the district management did not support the need for protected training time. Some FPs found the RTC sessions tiring after the day’s work. They had to prepare many topics, which they found overwhelming making it challenging to prepare them to the required standard:

‘Most times, we are overworked and tired because at the end of it, like, we’re just two, three of us, so at the end of the day you finish going through the whole day, from observed consultation of the patient, then by the time they come back in the afternoon, everybody is already tired’. (FP 3)

#### Registrars often feel unsupervised

According to most registrars, FP supervision in clinical and non-clinical contexts was less than adequate because of supervisor’s non-availability at clinics and RTCs. Family physicians were occupied with multiple responsibilities, such as consulting patients, teaching students and administrative duties. Some registrars expressed the need for more opportunities to observe supervisor consultations at clinics and especially at hospitals, as they can only perform a limited set of skills at the clinics:

‘I spent two months at PHC 10, three months at PHC 1. I got very little to no supervision in my primary health care. I want to say none. I hardly saw another doctor; it was a struggle for me just to see … I would love to have seen a doctor’. (RG 11)

#### Supervision is not standardised across the training sites

Supervision varied across the districts. For example, while four districts had academic sessions at RTC, FPs in one district felt that all training needs had to be covered in one clinic session, as they did not have academic sessions at RTC in their district. One of the FPs commented:

‘It’s not optimal as it is because we’re spending just one day in a whole week together. We are not able to cover everything we have to do. Because now we have to cover the training in terms of the observed consultations, we have to practice one or two topics, in that same day’. (FP 10)

Some FPs explained how limited exposure to other FPs in the same district hindered registrars’ access to varied expertise, skills and knowledge:

‘The other challenge is maybe the registrars are limited in getting the experience from other family physicians. Probably you are the one who’s going to train him, but previously when you compare, they will have different exposure, different skills, different approach, different knowledge, from a variety of family physicians’. (FP 2)

In some districts, FPs were not working alongside registrars. Yet, in other districts, the registrars worked with FPs in the same clinic for a short time, although there was no one-on-one allocation as an immediate supervisor. Family physicians felt that the objectivity of workplace-based assessments was reduced when the same supervisor was supervising the same registrar throughout their training years. Despite these site differences, the quality of supervision was good when one-on-one engagement took place, as described in the first two themes.

Another limitation identified by some of the FPs was their lack of skills across all domains and their preferences for teaching certain topics, which affected their one-on-one supervision:

‘You may be weak on women’s health, or you might be strong in paediatrics. Because it depends on where you came from, what you were doing before family medicine. So, I never have all the skills to help the registrar, but I’m just trying my best’. (FP 2)

### The training environment is challenging

Family physicians and registrars mentioned various challenges in the training environment, including resource shortages, heavy workloads and personal obligations, which affected training and learning opportunities.

#### Insufficient resources hinder training

Most FPs and registrars perceived problems related to a lack of human resources in the form of insufficient FPs, equipment, or space for consultations:

‘Some of the few challenges that we have in primary health care, that most of the skills that are listed we are not able to do them [*sic*] in the clinics. First of all, because of the laboratory issues. Secondly, most of the time, we don’t even have those resources. For instance, your LP needles are not there. Sometimes, we don’t even have injection needles. It’s [*sic*] a lot of issues’. (FP 10)

Registrars and FPs from remote sites felt that the shortage of FPs was more evident in these areas:

‘There are only two family physicians, so this district is quite big from … it’s 300 kms from the one side to the other side; it is difficult for two of them to get to all the registrars’. (RG 8)

Some registrars said that the lack of complex patients was why they could only practise a limited set of skills at the clinics, which meant that they were inadequately prepared for the national exit examinations:

‘The challenge for me is the complexity of the patients that we have here. So sometimes you feel that the reg [*registrar*] might seem to be doing well but because the patients are not … let me not say, are not difficult, like they are easy cases in a way, they are not that complex in terms of for … and I cannot say the … I cannot guarantee in terms of that, in the exam this is what the reg is going to get’. (FP 1)

#### Work–life balance is challenging for registrars

Most registrars perceived that the patient load was too heavy, which hindered training in their clinical contexts. As a result, they focused on seeing patients and clearing the queues instead of actual learning utilising the FM approach. These challenges reduced the opportunities for registrars to be supervised on their consultations and procedures:

‘You are faced with a challenge of service delivery versus (the) learning you need to learn [*sic*] as a registrar. So, obviously, that balance needs to be found where you can’t compromise service delivery for learning. And also, learning can’t be compromised at the expense of service delivery. So, it’s quite difficult to be in a busy clinic’. (RG 1)

Another challenge was managing personal and family obligations along with academic requirements. One of the registrars said:

‘You have a family as well when you come back home. You have children you have to take care of … and you also have your family medicine activities that you need to do, like your assignments, and you’re, should I say … things that have to be up to date, for your own training, but then you have other activities that require some time from you’. (RG 9)

#### The district hospitals are perceived as hostile

The perceived hostility towards FM training at some district hospitals posed a particular set of challenges. Some FPs felt that the managers in these hospitals were not welcoming or supportive of the training programme. The full-time MO felt threatened when FPs rotated at the hospitals to supervise the training of registrars. One of the FPs said:

‘Some of our colleagues are not favourably disposed to moving into the hospital … because of the hostility; it’s a two-pronged thing. Because of the hostility we’re also getting from DH4 [*district hospital*] because they always feel threatened when they see [*us*]’. (FP 3)

## Discussion

This study is the first attempt to explore the strengths and challenges of decentralised postgraduate FM supervision in an African context. Overall, participants perceived an acceptable level of postgraduate FM supervision at DCT sites, with good supervisor characteristics. Supervisors provided multiple training opportunities to registrars despite their numerous roles and responsibilities. Despite the several challenges that could impact the quality of supervision, many strengths of supervision were identified, which support the need for continuing DCT.

One of the significant strengths of supervision in this study was the individual engagement in the clinical context. Both FPs and registrars valued the informal training opportunities when they, for example, saw patients together, consulted on difficult and complex patients, and conducted in-depth discussions on diagnoses and management. The individual engagements facilitated good role-model behaviour and several types of interactions integral to supervision in WBL, including support seeking, monitoring, modelling, making meaning and feedback.^41^ The FP supervisors appreciated utilising mini-clinical evaluation exercises as formative rather than summative assessments during individual engagements, overcoming the tension between them, as described by Prentice et al (2020).^40^ The FP supervisors also valued the opportunities for creating learning experiences and providing feedback during one-on-one engagements with registrars. However, some registrars felt that these individual interactions were subjective and depended on the supervisors’ experience. They were dissatisfied with being supervised by junior supervisors or MO similar to the findings from studies evaluating FM programmes in other settings.^38,53^ In some districts, workplace-based assessments needed to be carried out by multiple assessors with different levels of experience in various contexts in order to assess registrar performance and make it more authentic.^37^ Registrars felt that mini-clinical evaluation exercises were sometimes rushed, resulting in minimal contact time with supervisors, as has been described previously.^54^ In other situations, the lack of pairing registrars with immediate supervisors hindered the possibility of assessing the registrar’s longitudinal progress in workplace-based assessments. The pairing of registrars with FPs, as seen in some districts, can cause leniency in workplace-based assessments because of the assessor–trainee relationship, as explained in a previous study.^40^

While fewer individual engagements were possible in non-clinical contexts, a positive aspect of supervision in these sites was receiving opinions and perspectives from multiple FPs, which the registrars felt improved their knowledge of specific topics. The senior registrars, particularly, benefitted from receiving different opinions, which provided a holistic picture of their progress.^40^

Another major strength was good clinical supervision by non-FP supervisors when registrars rotated in speciality disciplines, although it varied across departments. The non-FP supervisors engaged FM registrars to practise procedures repeatedly under their guidance, allowing them to master their skills. Despite its strengths, the non-FP supervision presented some challenges. While the focus of non-FP supervisors on clinical supervision was perceived as a positive aspect, the registrars felt that the non-FPs became absorbed in the clinical speciality, leaving insufficient time to practise the biopsychosocial aspects of patient consultation, which was aggravated by little or no FP supervision, as found previously.^55^ Although most non-FP supervisors were specialists, registrars from specialities other than FM and senior MO sometimes supervised FM registrars in some clinical departments. The lack of supervision training of these registrars and MO impacted their on-the-job training of the FM registrars. Previous studies in SA have shown that a lack of adequate training in supervision negatively influences the capability of supervision.^42,56^ Another aspect arising from the lack of training of the non-FP supervisors, mentioned by FPs and registrars, was the lack of understanding of FM outcomes and the scope of practice of registrars, which influenced the ability of registrars to incorporate FM principles or approaches in treating their patients. The lack of supervision and training challenges of registrars on FM concepts and topics by non-FP supervisors during their hospital rotations was also found in other settings.^38,57^ Some registrars described the need for constant engagement with non-FP supervisors and supervisory support visits by head of departments or academic training coordinators from the FM department to regional hospitals in order to address these challenges.

Registrars and FPs valued responsiveness to registrar learning needs by FPs as a strength. Family physician supervisors supported individual learners at the workplace, focussing on developing them and addressing their learning needs and potential, which is critical in DCT.^3,10^ According to registrars, they learnt different FM principles and their applications and sound FP approaches from their FP supervisors in all contexts. Family physicians felt that they were training registrars in all the required FP roles, such as clinical trainer, community leader and governance roles, other than their consultant role, although registrars did not perceive that. As registrars did not recognise these FP roles, this suggests the need for creating more registrar opportunities to practise all the expected FP roles^35,42^ in order to ensure that all learning outcomes^37^ are achieved.

According to registrars and FPs, FP supervisors had good clinical trainer and supervisor attributes or acted as good role models with excellent communication and organisational skills, openness, honesty, willingness to teach and teamwork, as found in previous studies.^12,24,25,58^ In some districts, FP supervisors worked as a team when challenged because of their multiple roles or level of experience in training by sharing their responsibilities. Teamwork is essential for professional satisfaction, as described in the UK medical education system.^39^ Developmental conversations^20^ between supervisors and registrars become ideal when the learning environment and supervisor–registrar relationship is optimised,^26^ which were constrained in some training sites. Supervisors need to strive to be good role models in the workplace as part of learning organisations to develop and maintain good communities of practice^13^ that influence registrar learning experiences and professional identity formation.

Some FPs perceived a lack of professional behaviour of registrars as an obstruction to meaningful engagement. However, teaching and assessing professionalism should not be left to chance. Professional attributes should be intentionally taught and regularly assessed among registrars.^21,59^ When the registrar’s performance is affected, the FP’s responsibility is to explore the factors affecting it, including the registrar’s professional behaviour. Multiple opportunities exist to explore these factors on a DCT platform, where FPs and registrars work together. An approach to the registrar’s inadequate performance is to explore registrars’ skills and knowledge level and internal factors (such as personality traits or attitudes determining their professionalism) and external factors (such as workload, resource availability and family responsibilities).^22^ The selection of appropriate candidates with adequate baseline skills and knowledge was also a critical factor identified by Ras et al (2020).^45^ This is because, in WBL, supervision depends substantially on self-regulated learning,^28^ registrars’ reflection capabilities and how they utilise the informal learning opportunities in workplace.

Most participants agreed that the severe shortages in human resources, equipment and infrastructure, and inadequate exposure to complex patients and skills impacted on registrar’s learning in primary-care clinical contexts. Most registrars felt that the supervision was not always ideal in clinical and non-clinical contexts because of inadequate supervision or FPs’ heavy workloads. Many registrars expressed the desire for supervision to be more visible and to have supervisors in their environment more often. The non-availability or inadequate number of FPs resulted in registrars competing for interactions or contact time, which affected their training opportunities. The emphasis was on the need for more modelling opportunities with their supervisors, as identified in several previous studies.^44,45,40^

Most FPs struggled to fulfil their multiple roles, including administrative tasks, service delivery and training commitments in both clinical and non-clinical contexts. Effective supervisors are expected to fulfil various roles and responsibilities, such as medical experts, managers, communicators, healthcare advocates and scholars in clinical settings.^60^ Family physicians felt the need for protected time for registrars and supervisors to fulfil training requirements while busy with their various roles and responsibilities and management support, as found in a United Kingdom study evaluating WBL.^61^ The concepts of supervision in WBL emphasis informal learning and reflection, need to be explained to registrars but supervisors should provide these opportunities, allow registrars to work in other communities of practice,^13^ maximise cognitive apprenticeship strategies,^11^ create a culture of belonging and set aside time for engagement.^10^ This can avoid the disappointment of the registrar expectations of having more formal sessions in postgraduate training. The FP supervisors should also be clinically relevant and competent,^19,44^ which was not the case here, as FPs identified a lack of teaching skills in some clinical domains.

Another constraint affecting the quality of supervision was the training environment itself. An enabling training environment in DCT depends not only on the FP training abilities but also on the workplace infrastructure, organisational structure, equipment, optimum supervisor-to-trainee ratio and appropriate patient mix,^3,58^ which was a limitation at most sites. Contextual health system factors, such as workload, work patterns and distribution, were major system factors that influenced learning interactions between supervisors and registrars, as identified in previous studies.^7,10,45,41^ In smaller clinics, the training environment was even worse.^7,27^ In addition, few registrars recognised the need for developing coping mechanisms to balance their academic requirements with family and work commitments, which impacted negatively their training opportunities.

Even though the need for standardisation of DCT programmes across universities was identified in previous studies in SA,^58^ it remains a challenge as observed by both groups of participants in this study. The perceived hostility from some district hospitals not identifying themselves as training institutions also needs to be addressed in order to standardise training opportunities for registrars across these hospitals.

### Limitations

The principal author interviewed the participants, being the supervisor for three of the 11 registrars and a colleague of the FPs. This could have affected the participants’ responses and introduced bias. However, the benefits of being a researcher with insider knowledge outweighed the risks.^62^ Electronic interviews with some participants may have missed some non-verbal cues and responses, as there was no face-to-face contact.

### Recommendations

The university needs to play an advocacy role in improving the supervision and support given to registrars, achieving standardisation, and addressing the human and other resource shortages across the decentralised training sites. There should be a provision of protected time both for registrars and especially for supervisors, who are busy with multiple roles and responsibilities approved by the district management. Regular conversations between the various sites on improving supervision, training and support for registrars in clinical contexts such as clinics, hospitals and RTCs are essential. Multiple supervisors should carry out workplace-based assessments in different contexts to measure registrar performance authentically in order to improve their learning experience. Regular updating of FPs’ skills and faculty training in supervision of both FPs and other non-FP supervisors involved in training are required to promote alignment with registrars’ learning needs. Registrars should be adequately educated and prepared on aspects of WBL in decentralised contexts.

## Conclusion

This study exploring postgraduate FM supervision in a decentralised training context identified multiple strengths and challenges. While there is a good level of supervision, it is context-specific and supervisor-dependent found in other studies. Supervisors displayed good clinical-teacher characteristics, but their supervisory roles can be further improved in various contexts. Good supervision of registrars requires excellent clinical knowledge, professionalism and allows internalising appropriate FP approaches and skills. However, there are supervisory challenges because of multiple FP roles, a lack of standardisation and insufficient understanding of the registrar’s scope of practice by non-FP supervisors. The training environment has various other challenges, such as heavy workload and human resource shortages. The study findings provide a baseline knowledge of aspects of postgraduate supervision and its strengths and challenges in similar contexts. Future research involving other decentralised training sites in sub-Saharan Africa will provide a more comprehensive picture of postgraduate FM supervision. This enhances the training of FPs based on primary care contexts, addressing the community’s needs and translating into better health outcomes.

## Appendix 1: Interview guide

Questionnaire A (Registrar)

1. How are you supervised by Family Physicians at
  - Regional training centre
  - Primary health care clinics and
  - During skills rotations in the district and regional hospitals?
2. What are the strengths and challenges in supervision at
  - Regional training centre,
  - Primary care clinics and
  - At skills rotations in the district and regional hospitals?
3. How are you supervised by other specialists at the district and regional hospitals during your skills rotations?
4. What are the strengths and challenges of supervision during skills rotations in the district and regional hospitals?

Questionnaire B (Family Physician)

1. How do you supervise registrars at
  - Regional training centre
  - Primary care clinics and
  - During skills rotations in the district and regional hospitals?
2. What are the strengths and challenges in your supervision at
  - Regional training centre
  - Primary care clinics and
  - In the district and regional hospitals?
3. How are the registrars supervised by other specialists at the district and regional hospital during their skills rotations?
4. What are the strengths and challenges of supervision during skills rotations at district and regional hospitals?
